# High-pH reversed-phase fractionated neural retina proteome of normal growing C57BL/6 mouse

**DOI:** 10.1038/s41597-021-00813-1

**Published:** 2021-01-26

**Authors:** Ying Hon Sze, Qian Zhao, Jimmy Ka Wai Cheung, King Kit Li, Dennis Yan Yin Tse, Chi Ho To, Thomas Chuen Lam

**Affiliations:** 1 Laboratory of Experimental Optometry, Centre for Myopia Research, School of Optometry, Hong Kong Polytechnic University, Hong Kong, China; 2 State Key Laboratory of Chemical Biology and Drug Discovery, Department of Applied Biology and Chemical Technology, Hong Kong Polytechnic University, Hong Kong, China; 3 Centre for Eye and Vision Research, Hong Kong, China

**Keywords:** Retina, Proteomics

## Abstract

The retina is a key sensory tissue composed of multiple layers of cell populations that work coherently to process and decode visual information. Mass spectrometry-based proteomics approach has allowed high-throughput, untargeted protein identification, demonstrating the presence of these proteins in the retina and their involvement in biological signalling cascades. The comprehensive wild-type mouse retina proteome was prepared using a novel sample preparation approach, the suspension trapping (S-Trap) filter, and further fractionated with high-pH reversed phase chromatography involving a total of 28 injections. This data-dependent acquisition (DDA) approach using a Sciex TripleTOF 6600 mass spectrometer identified a total of 7,122 unique proteins (1% FDR), and generated a spectral library of 5,950 proteins in the normal C57BL/6 mouse retina. Data-independent acquisition (DIA) approach relies on a large and high-quality spectral library to analyse chromatograms, this spectral library would enable access to SWATH-MS acquisition to provide unbiased, multiplexed, and quantification of proteins in the mouse retina, acting as the most extensive reference library to investigate retinal diseases using the C57BL/6 mouse model.

## Abstract (summary)

**Table:** 

Measurement(s)	retina
Technology Type(s)	mass spectrometry
Sample Characteristic - Organism	Mus musculus

Machine-accessible metadata file describing the reported data: 10.6084/m9.figshare.13128044

## Background & Summary

The retina is the site of many posterior ocular diseases, including retinal detachment^1^, diabetic retinopathy (DR)^2^, age-macular degeneration (AMD)^3^, glaucoma^4^, and myopia^5^. It can be divided into primarily ten major layers including (1) inner limiting membrane (ILM); (2) nerve fiber layer; (3) ganglion cell layer; (4) inner plexiform layer (IPL); (5) inner nuclear layer (INL); (6) outer plexiform layer (OPL); (7) outer nuclear layer; (8) outer limiting membrane (OLM); photoreceptor layer (PL); and (10) retinal pigmented epithelium (RPE) monolayer^6^. The C57BL/6 mouse is the most commonly used inbred strain for research, which has the advantage of its genome being sequenced, as well as the permissive genetic background allows maximal expression of most mutations. Jeon el al (1998) reported seven major cell populations in the C57BL/6 mouse retina, including rod photoreceptors, cone photoreceptors, Müller glia cells, retinal ganglion cells (RGC), horizontal cells, amacrine cells, and bipolar cells^7^. Modern transcriptomics analysis has recently revealed 39 transcriptionally distinct cell populations using single-cell RNA sequencing (scRNA-seq), supporting the presence of novel candidate cell subtypes of microglia, retinal endothelial cells, and astrocytes recently^8^. In addition to the wild-type mouse (*Mus musculis*), other well-established mouse models include the retinal degeneration 10 (rd10) mutant is used to study neuronal degeneration of the retina^9^ and Ins2Akita for research into early retinal complications in diabetes^10^. The study of retinal diseases has been limited by a lack of retinal cell lines comparable to the neural retina, with only limited provision of the RPE^11,12^ (D407, ARPE19) and RGC^13^ (RGC-5) cells. Therefore, profiling normally growing C57BL/6 mouse retinal proteins could provide an important reference dataset to advance the understanding of retinal physiology and its ocular functions. A similar approach has been applied in for guinea pig^14^, human^15^ and zebrafish^16^ tissues in recent years. In particular, supplementary to the analysis of nine C57BL/6 mice tissue samples (brain, gallbladder, pancreas, large intestine, small intestine, liver, lung, stomach, and urinary bladder) were reported with the total of 11,340 proteins, acquired in 180 samples, comprises of 437 DDA scans^17^.

Highly sensitive mass spectrometry has become an indispensable tool to investigate protein expression, interaction, and post-translational modification (PTM). Data-dependent acquisition (DDA) extracts the most abundant eluted parent ions of a survey scan (MS1) and subsequently these are fragmented in a collision compartment (MS2) to enable peptide sequencing and identification by software data processing. The biased extraction affected by protein abundance has hindered reproducible quantification between sample runs. The generation of a spectral library assists the construction of a Data-independent acquisition (DIA) approach, in particular, a sequential windowed acquisition of all theoretical fragment ion mass spectra (SWATH-MS) that allows reproducible and precise quantification of thousands of proteins in complex tissue^18^. New biological insights have been made possible by combination and reutilization of open-access retinal spectral libraries for interrogation by SWATH‐MS recently^19^.

**Fig. 1:**
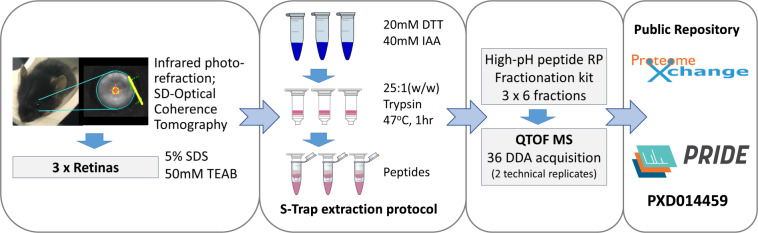
Experiment design and sample preparation workflow.

Here we present the most comprehensive DDA spectral library, compiled using offline high-pH fractionation, which contains 5,950 non-redundant proteins, (54,865 peptides) at 1%FDR of the C57BL/6 mouse retina, accounting for 35% of the total reviewed proteins listed in the UniProt protein database (Mus musculus, UP000000589). The library was generated by combining the results of DDA from a total of 38 injections, with 36 peptide samples and two pooled samples extracted from normal adult mouse retinas. (Fig. 1 and Table 1) The dataset was generated with the combined sample preparation protocol of suspension-trap, S-Trap^20^, and subsequently with the commercially available high-pH fractionation kit that allows ultrafast reproducible lysis, digestion, and fractionation of the neural retina. This submission provides the reference list of proteomes identified *in-vitro* in C57BL/6 mouse neural retina for ocular proteomics research. The generated data has been accepted by the PRIDE repository^21^ for open access.

**Table 1:** Sample annotation, fractions and acquired peptide amount.

PRIDE File No.	Retina No.	Sample Type	Fraction	Data Type	Technical Replicate	Injection Volume (ul)	Peptide Amount (ug)
1	R1	Retina	F1	DDA	T1	4	2
2	R2	Retina	F1	DDA	T1	4	2
3	R3	Retina	F1	DDA	T1	4	2
4	R1	Retina	F2	DDA	T1	6	1.8
5	R2	Retina	F2	DDA	T1	6	1.8
6	R3	Retina	F2	DDA	T1	6	1.8
7	R1	Retina	F3	DDA	T1	6	1.8
8	R2	Retina	F3	DDA	T1	6	1.8
9	R3	Retina	F3	DDA	T1	6	1.8
10	R1	Retina	F4	DDA	T1	6	1.8
11	R2	Retina	F4	DDA	T1	6	1.8
12	R3	Retina	F4	DDA	T1	6	1.2
13	R1	Retina	F5	DDA	T1	6	1.5
14	R2	Retina	F5	DDA	T1	6	1.5
15	R3	Retina	F5	DDA	T1	6	1.5
16	R1	Retina	F6	DDA	T1	6	1.2
17	R2	Retina	F6	DDA	T1	6	1.2
18	R3	Retina	F6	DDA	T1	6	1.2
25	R1	Retina	F1	DDA	T2	4	2
26	R2	Retina	F1	DDA	T2	4	2
27	R3	Retina	F1	DDA	T2	4	2
28	R1	Retina	F2	DDA	T2	6	1.8
29	R2	Retina	F2	DDA	T2	6	1.8
30	R3	Retina	F2	DDA	T2	6	1.8
31	R1	Retina	F3	DDA	T2	6	1.8
32	R2	Retina	F3	DDA	T2	6	1.8
33	R3	Retina	F3	DDA	T2	6	1.8
34	R1	Retina	F4	DDA	T2	6	1.8
35	R2	Retina	F4	DDA	T2	6	1.8
36	R3	Retina	F4	DDA	T2	6	1.2
37	R1	Retina	F5	DDA	T2	6	1.5
38	R2	Retina	F5	DDA	T2	6	1.5
39	R3	Retina	F5	DDA	T2	6	1.5
40	R1	Retina	F6	DDA	T2	6	1.2
41	R2	Retina	F6	DDA	T2	6	1.2
42	R3	Retina	F6	DDA	T2	6	1.2
49	Pool (R1-R3)	Retina	—	DDA	T1	4	2
50	Pool (R1-R3)	Retina	—	DDA	T2	4	2

## Methods

### Animals

Mice were maintained as in-house breeding colonies at the centralised animal facility, The Hong Kong Polytechnic University. Wild-type C57BL/6 mice (n = 3) were refracted between 3 and 6 weeks of age to assess refractive development under unmanipulated visual conditions. The eye of each animal was examined at postnatal day 25 with a high resolution spectral-domain optical coherence tomography (SD-OCT) to confirm the physiology of the external and internal ocular structures was normal. Animals were housed in standard mouse cages at 25 °C on a schedule of 12:12 hour of light/dark cycle, with mouse pellets and water available *ad libitum*. Researchers were licenced by the Department of Health, HKSAR government and all procedures performed in this study received ethics approval from the Animal Subjects Ethics Sub-Committee (ASESC), The Hong Kong Polytechnic University and complied with the Association of Research in Vision and Ophthalmology (ARVO) statement for the use of animals in ophthalmology and vision research.

### Refractive error measurement with Infrared photometer

Refractive error was measured with a customised infrared photorefractor (Steinbeis Transfer Centre, Germany) as previously described^22,23^. In brief, the pupil of each eye was dilated with mydrin-P ophthalmic solution, containing 0.5% tropicamide and 0.5% phenylephrine HCl for 15 minutes. The mouse was sedated (ketamine 70 mg/kg; xylazine 10 mg/kg delivered by intraperitoneal injection) and then placed on the SD-OCT cylindrical platform, the distance from the camera being based on the image acuity. The mouse eye was aligned to its Purkinje image with gaze control in the x- and y-axes smaller or equal to 5. The software automatically collects 99 datapoints within the gaze control, repeated for three technical replicates. The refractive error is represented as the mean value in diopters (D).

### Ocular dimension measurement using SD-OCT

The axial length and the dimension of each ocular component was analysed by a SD-OCT system (Envisu R4310, Leica, Germany) with a 50° mouse probe after refractive error measurement. First, the mouse was aligned and positioned at a close distance to the probe under free scan mode. The optical disc position was Identified and adjusted to a position 2 mm above the optic nerve. This distance allowed the display of the whole eye structure from the corneal apex to the choroidal sclera layer. The eye was scanned in radial volume mode (A-Scans = 1000 lines, B-Scans = 6 scans, 32 frames, 80 lines of inactive A-scans, 0.4 mm diameter), which was repeated to obtain three technical replicates. The length of each component is represented as its mean value in mm. Axial length (AXL) was defined as the distance from the corneal apex to the posterior retina^24^. Corneal thickness (CT) was measured from the corneal apex to the posterior of cornea. Anterior chamber depth (ACD) was measured from the posterior of the cornea to anterior of the lens. Lens thickness (LT) was measured up to posterior lens. Vitreous chamber depth (VCD) was measured up to the retinal nerve fiber layer. Retinal thickness (RT) was measured up to retinal pigment epithelial layer.

### Retina extraction

Procedures employed were similar to published methods with minor modification^25^. Mice were sacrificed with cervical dislocation after *in-vivo* ocular measurement. Both eyes were enucleated and immediately stored in ice-cold phosphate-buffered saline (PBS). The cornea was separated after hemisecting the eye equatorially, the crystal lens was removed from the eye cup with forceps. The remaining tissue was submerged in ice-cold PBS in a cell culture dish. The tissue was shaken gently, with the forceps holding the posterior sclera only. The retinal layer was detached from the retinal pigmented epithelium (RPE) and choroidal-sclera compartment. The retina was rinsed with ice-cold PBS, transferred to a 1.7 mL Eppendorf tube and snap-frozen in liquid nitrogen immediately.

### Mouse retinal protein extraction

Three normal retinas from different individuals were homogenised in a liquid nitrogen cooled tissue homogenizer (Precellys Evolution, Bertin Instruments, France) with 150 μL of lysis buffer containing 5% sodium dodecyl sulfate (SDS) and 50 mM triethylammonium bicarbonate (TEAB). The sample was homogenised at 4 °C, 7,000 rpm for 30 s x 4 cycles with 20 s intervals in the homogenisation tube (CKMix, Precellys Lysing Kit). The sample was briefly centrifuged, the soluble retina lysate recovered and centrifuged at 15,000 rpm for 30 min at 4 °C. The supernatant was collected, and the bicinchoninic acid assay (Pierce Rapid Gold BCA Protein Assay, A53225, Thermo Fisher) to determine the protein concentration of each sample.

### Sample preparation

Aliquots of retinals lysate (200 ug) were reduced with 200 mM dithiothreitol (DTT) at 95 °C for 10 min and then alkylated with 400 mM iodoacetamide (IAA) at room temperature in the dark for 10 min. The SDS lysate was acidified with 12% aqueous phosphoric acid, 1:10 (v/v) to a final concentration of 1.2%. The solution was then diluted with the S-Trap protein binding buffer (90% methanol, 0.1 M TEAB, pH 7.55), 6:1 (v/v, S-Trap: total vol.). The mixture was transferred to the S-Trap micro spin column and centrifuged at 4,000 g for 20 s. The captured protein was washed with 150 μL S-Trap protein binding buffer and the centrifugation steps repeated three times. Protein digestion step was performed using 20 μL of digestion buffer containing trypsin, 1:25 (w/w, trypsin: protein) added to the top of the micro column and was stored at 47 °C for 1 hour. Peptides were eluted in three sequential steps: (1) 40 μL of 50 mM TEAB; (2) 40 μL of 0.2% aqueous formic acid: and (3) 35 μL of 50% acetonitrile, 0.2% formic acid. The three eluents were pooled and dried with vacuum centrifuging at 4 °C (Labconco, Kansas City, MO, USA). Peptides were re-suspended with 20 μL of 0.1% formic acid (FA) for fractionation.

### High-pH reserved phase peptide fractionation

Peptides (100 ug) were diluted to 300 μL with 0.1% trifluoroacetic acid. Fractionation was performed as described in the manufacturer’s manual (Cat No. 84868, Thermo Fisher Scientific). In brief, retina samples were fractionated with 6 increments (10, 12.5, 15, 17.5, 20 and 22.5%) of acetonitrile (ACN) in 0.1% TFA. These eluents were dried with vacuum centrifuge at 4 °C. Peptide concentration was measured using colorimetric peptide assay (Cat No. 23275, Thermo Fisher Scientific). Peptide samples were normalised to 0.5 ug/μL with 0.1% FA in water for mass spectrometric analysis.

### DDA acquisition of retina samples

Peptides were loaded with isocratic loading buffer (0.1% FA, 5% ACN in water) to a reversed-phase chromatography trap-column (C18, 350 um x 0.5 mm) at 2 μL/min for 15 min. Peptides were separated by a C18 reversed-phase analytical column (Acclaim PepMap 100, C18, 100 Å, 5 um, 100 um x 30 cm) at a flow rate of 350 nl/min with the following gradient: 0–0.5 min: 5% B (98% ACN, 0.1% FA), 0.5–90 min: 10% B, 90–120 min: 20% B, 120–130 min: 45% B, 130–135 min: 45% B, 135–141 min: 80% B and 20% buffer A (2% ACN, 0.1% FA), 141–155 min: 5% B. Peptides were introduced into the mass spectrometer (Sciex TripleTOF 6600) with a SilicaTip electrospray emitter (New Objective, FS360-20-10-N-20-C12, 10 um) handled by nano-flow liquid chromatography (NanoLC 415, Eksigent). The MS1 spectra were obtained from 350 to 1800 m/z. The top 50 ions exceeding the threshold of 125 cps, charge state from +2 to +5 in each cycle, were selected using an accumulation time of 250 ms with a dynamic exclusion time of 18 s. Precursor peptides were fragmented by collision induced dissociation (CID) with rolling collision energy for peptides charge 2+, a collision energy spread of 15 eV was added.

### Generation of SWATH spectral library

Raw data files from all the fractions were analysed using ProteinPilot 5.0.1 software (SCIEX, US). *Mus musculus (Mouse)* proteome database, containing 17,015 of reviewed proteins, was acquired from the UniProt proteome dataset UP000000589. Trypsin was set as the enzyme for digestion, Carbamidomethylation was set for fixed modification with all possible biological modifications selected. FDR analysis was performed by searching MS/MS spectra against a given target database combined with a reserved-sequence decoy proteome database. Peptide samples were injected between 4 to 6 µL, ranging from 1.2 to 2 µg due to varied peptide concentration (Table 1).

## Data Records

The raw mass spectrometry readout DDA files were generated with Sciex proprietary software in (.wiff) format for library generation. The converted XML files, consensus spectral library, and ProteinPilot group file containing protein identification (ProteinPilot version 5.0.1) have been deposited to the ProteomeXchange Consortium via the PRIDE partner repository^26^.

## Technical Validation

### Validation of ocular parameters in normal C57BL/6 mouse

**Fig. 2:**
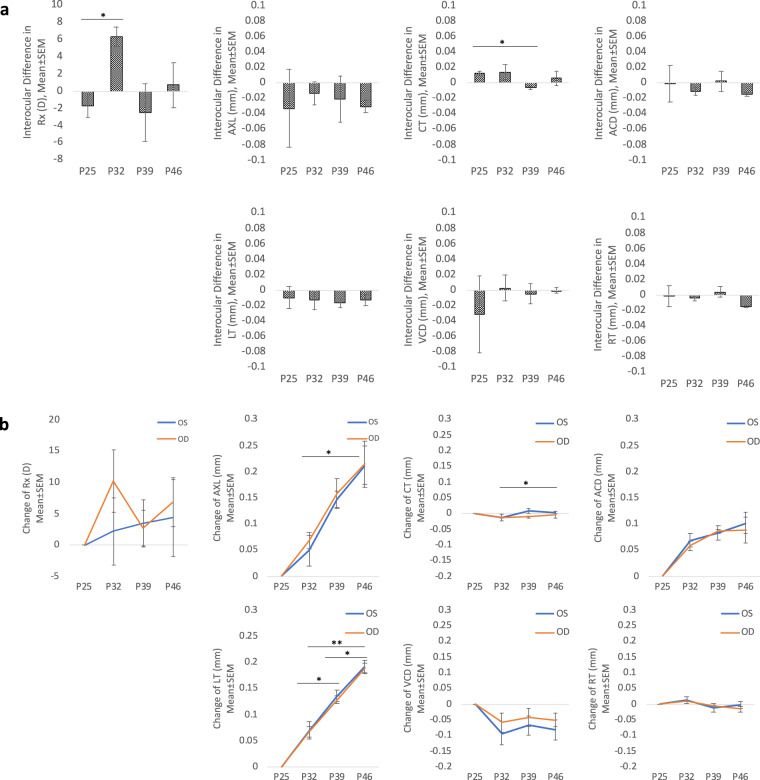
Ocular dimensions in normal growing C57BL/6 mice between postnatal day 25 and 46. Changes in ocular structure in normal growing C57BL/6 in Rx, AL, CT, ACD, LT, VCD and RT. **(a)** Interocular difference in each component in P25, P32, P39 and P46. Significant intraocular difference in refraction in P32 and corneal thickness on P39 (n = 3, p < 0.05). **(b)** Significant relative elongation in axial length (mm) and lens thickness (mm) in all time-points (n = 3, *p < 0.05; **p < 0.01).

The nocturnal mouse model has the potential to become an important model for studies of genetic and proteome alteration in the control of eye growth and myopia. The proteome dataset was built on the background of un-touched, normal C57BL/6 mice recovered at postnatal age (days) P46. Biometric measurements were performed at age P25, P32, P39, and P46 respectively. The dimensions in each ocular component between age 25 and 46 are shown in Table 2. Interocular differences in each ocular component, corneal thickness, anterior chamber depth, lens thickness, vitreous chamber depth, and retinal thickness are shown in Fig. 2. The axial length growth and lens thickness could be described by linear regressions. The observed constant growth rate in C57BL/6 mouse was similar to that previously reported^27^. The observed axial length of about 2.9 mm on day 22, was similar that reported by a study determining values from age 22 to 100 d in C57BL/6 mice^28^. Despite similar axial lengths, there were noticeable differences in the distribution of other components, with thicker corneal layer (0.0887 mm on P21) and increased anterior chamber depth (0.2854 mm on P21). However, there were no statistically significant differences in axial length, corneal thickness, anterior chamber depth, lens thickness, vitreous chamber depth, retinal thickness from age 25 to 46. The interocular difference was calculated by averaging and subtracting the dimension in the contralateral eye on the same time-point.

**Table 2:** Dimension in ocular compartments between age 25 and 46. Rx = refraction in diopters; AXL = axial length (mm); CT = corneal thickness (mm); ACD = anterior chamber depth (mm); LT = lens thickness (mm); VCD = vitreous chamber depth (mm); RT = retinal thickness (mm).

Age	P25	P32	P39	P46
**Rx (D)**	−3.2 ± 2.5	3.1 ± 7.6	−0.1 ± 4	2.4 ± 6.4
**AXL (μm)**	3066 ± 62	3124 ± 38	3218 ± 35	3279 ± 21
**CT (μm)**	98 ± 10	85 ± 15	96 ± 8	98 ± 9
**ACD (μm)**	260 ± 20	324 ± 9	344 ± 16	355 ± 28
**LT (μm)**	1779 ± 19	1848 ± 19	1911 ± 15	1969 ± 20
**VCD (μm)**	755 ± 50	680 ± 23	701 ± 19	689 ± 8
**RT (μm)**	181 ± 11	192 ± 9	172 ± 10	172 ± 13

Axial length equal to the sum of each ocular compartments (CT, ACD, LT, VCD, RT). Measurements were represented as the mean value of left and right eyes in mm, ± SD (n = 6).

### Characteristics of the proteomics dataset

**Fig. 3:**
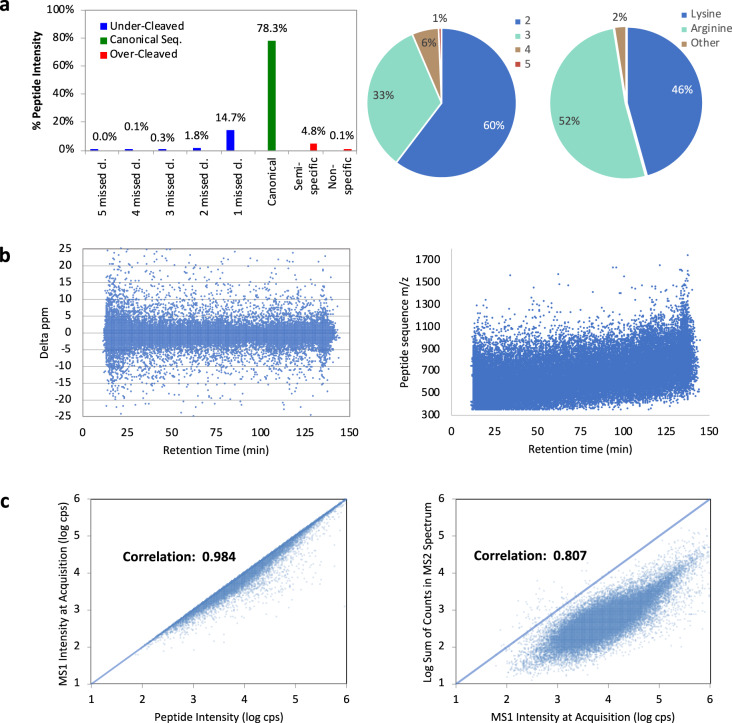
Statistical analysis of the proteomics data. (**a**) Enzymatic digestion efficiency with 78.2% of expected canonical sequence peptides, 5% over-cleaved sequence peptides, and 15.8% under-cleaved sequence peptides; the distribution of peptide precursor charge state with 60% doubly charged, decreasing to 5+ accordingly. The tryptic digestion resulted in uncontrolled C-terminal cleavage with 46% and 52% devoted to lysine and arginine respectively. **(b)** Precursor mass error in ppm during acquisition. The identified peptide sequence mass-to-charge value and its retention time in C18 chromatographic separation. (**c**) The correlation of MS1 intensity with good correlation to identified peptide intensity and good correlation between MS2 signals to MS1 intensity, showing the robustness of the mass spectrometry system in ion transmission and corresponding confidence on MS2 peptide mass fingerprint spectrum.

**Fig. 4:**
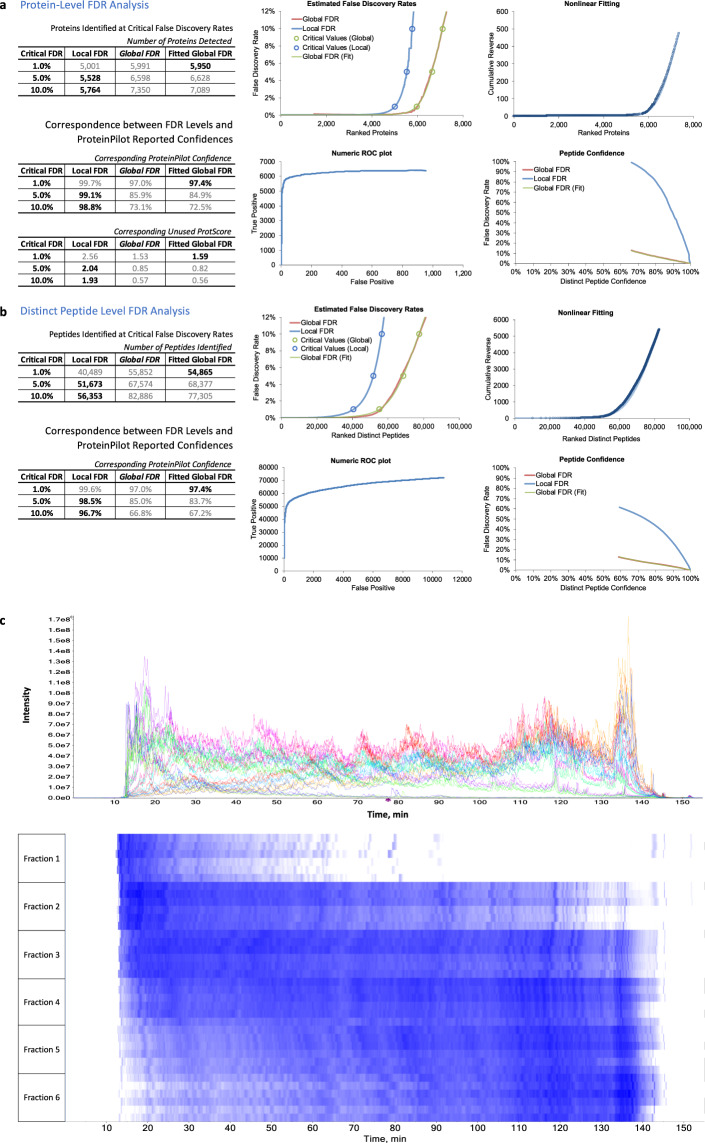
False discovery rate control and TIC chromatogram. False discovery rate (FDR) cut-off at 1%, 5%, and 10% confidences, nonlinear fitting of racked proteins, ROC plot of identification and estimated FDR in **(a)** protein and **(b)** peptide level. At 1%FDR cut-off, the dataset identified 5,950 proteins and 54,865 peptides. **(c)** Total ion chromatogram and heatmap of ion intensity acquired in a 120-minute gradient separation. From individual analysis of fraction 1 to 6 identified 2721, 4325, 4800, 4391, 3847, and 3766 unique proteins at 1% FDR respectively.

The combination of S-Trap protein extraction and high-pH peptide fractionation procedures allowed for rapid and reproducible sample preparation of mouse retinas. This dataset represents the first S-Trap application of an established protocol of retinal sample for biological investigation using SWATH-MS approach. The overall characteristics of the generated DDA spectral library are listed in Fig. 3. The tryptic digestion yielded uncontrolled C-terminal cleavage with 46% and 52% devoted to lysine (K) and Arginine (R) amino acids. The abundance of peptide charge ranges from +2 charge to +5, with the majority (60%) of peptides found in +2 charge state. The instrument has been maintained for high mass accuracy and positive correlation in ion intensity in MS1 and MS2. The false discovery rate (FDR) was controlled at 1% in protein and peptide level using ProteinPilot (SCIEX, US), the stringent community standard in proteomics assay (Fig. 4). This high confidence retina proteome dataset presents the first, and the largest DDA spectral library, at the time of submission, for eye and vision research using the popular C57BL/6 mouse model.

## References

[1] Ghazi NG, Green WR (2002). Pathology and pathogenesis of retinal detachment. Eye (Lond).

[2] Feit-Leichman RA (2005). Vascular damage in a mouse model of diabetic retinopathy: relation to neuronal and glial changes. Invest. Ophthalmol. Vis. Sci..

[3] Ethen CM, Reilly C, Feng X, Olsen TW, Ferrington DA (2006). The proteome of central and peripheral retina with progression of age-related macular degeneration. Invest. Ophthalmol. Vis. Sci..

[4] Tezel G (2013). A proteomics view of the molecular mechanisms and biomarkers of glaucomatous neurodegeneration. Prog. Retin. Eye Res..

[5] Yu, F. J. *et al*. Alteration of retinal metabolism and oxidative stress may implicate myopic eye growth: Evidence from discovery and targeted proteomics in an animal model. *J. Proteomics*, 103684 (2020). 10.1016/j.jprot.2020.103684 32061809

[6] Willermain F (2014). Origins and consequences of hyperosmolar stress in retinal pigmented epithelial cells. Front. Physiol..

[7] Jeon CJ, Strettoi E, Masland RH (1998). The major cell populations of the mouse retina. J. Neurosci..

[8] Macosko EZ (2015). Highly Parallel Genome-wide Expression Profiling of Individual Cells Using Nanoliter Droplets. Cell.

[9] Gargini C, Terzibasi E, Mazzoni F, Strettoi E (2007). Retinal organization in the retinal degeneration 10 (rd10) mutant mouse: a morphological and ERG study. J. Comp. Neurol..

[10] Barber AJ (2005). The Ins2Akita mouse as a model of early retinal complications in diabetes. Invest. Ophthalmol. Vis. Sci..

[11] Carr AJ (2011). The expression of retinal cell markers in human retinal pigment epithelial cells and their augmentation by the synthetic retinoid fenretinide. Mol. Vis..

[12] Dunn KC, Aotaki-Keen AE, Putkey FR, Hjelmeland LM (1996). ARPE-19, a human retinal pigment epithelial cell line with differentiated properties. Exp. Eye Res..

[13] Van Bergen NJ (2009). Recharacterization of the RGC-5 retinal ganglion cell line. Invest. Ophthalmol. Vis. Sci..

[14] Shan SW (2018). Integrated SWATH-based and targeted-based proteomics provide insights into the retinal emmetropization process in guinea pig. J. Proteomics.

[15] Rosenberger G (2014). A repository of assays to quantify 10,000 human proteins by SWATH-MS. Sci. Data.

[16] Blattmann P (2019). Generation of a zebrafish SWATH-MS spectral library to quantify 10,000 proteins. Sci. Data.

[17] Zhong CQ (2020). Generation of a murine SWATH-MS spectral library to quantify more than 11,000 proteins. Sci. Data.

[18] Collins BC (2017). Multi-laboratory assessment of reproducibility, qualitative and quantitative performance of SWATH-mass spectrometry. Nat. Commun..

[19] Palmowski P (2019). The Generation of a Comprehensive Spectral Library for the Analysis of the Guinea Pig Proteome by SWATH-MS. Proteomics.

[20] HaileMariam M (2018). S-Trap, an Ultrafast Sample-Preparation Approach for Shotgun Proteomics. J. Proteome Res..

[21] Perez-Riverol Y (2019). The PRIDE database and related tools and resources in 2019: improving support for quantification data. Nucleic Acids Res..

[22] Pardue MT (2008). High susceptibility to experimental myopia in a mouse model with a retinal on pathway defect. Invest. Ophthalmol. Vis. Sci..

[23] Schaeffel F (2008). Test systems for measuring ocular parameters and visual function in mice. Front. Biosci..

[24] Zhou X (2008). Biometric measurement of the mouse eye using optical coherence tomography with focal plane advancement. Vision Res.

[25] Yu FJ (2017). Isotope-coded protein label based quantitative proteomic analysis reveals significant up-regulation of apolipoprotein A1 and ovotransferrin in the myopic chick vitreous. Sci. Rep..

[26] Lam TC (2020). PRIDE.

[27] Zhou G, Williams RW (1999). Mouse models for the analysis of myopia: an analysis of variation in eye size of adult mice. Optom. Vis. Sci..

[28] Schmucker C, Schaeffel F (2004). A paraxial schematic eye model for the growing C57BL/6 mouse. Vision Res..

